# Shaky Student Growth? A Comparison of Robust Bayesian Learning Progress Estimation Methods

**DOI:** 10.3390/jintelligence10010016

**Published:** 2022-03-01

**Authors:** Boris Forthmann, Natalie Förster, Elmar Souvignier

**Affiliations:** Institute of Psychology in Education, University of Münster, 48149 Münster, Germany; natalie.foerster@uni-muenster.de (N.F.); elmar.souvignier@uni-muenster.de (E.S.)

**Keywords:** progress monitoring, Bayesian analysis, slope, growth, robust estimation

## Abstract

Monitoring the progress of student learning is an important part of teachers’ data-based decision making. One such tool that can equip teachers with information about students’ learning progress throughout the school year and thus facilitate monitoring and instructional decision making is learning progress assessments. In practical contexts and research, estimating learning progress has relied on approaches that seek to estimate progress either for each student separately or within overarching model frameworks, such as latent growth modeling. Two recently emerging lines of research for separately estimating student growth have examined robust estimation (to account for outliers) and Bayesian approaches (as opposed to commonly used frequentist methods). The aim of this work was to combine these approaches (i.e., robust Bayesian estimation) and extend these lines of research to the framework of linear latent growth models. In a sample of *N* = 4970 second-grade students who worked on the quop-L2 test battery (to assess reading comprehension) at eight measurement points, we compared three Bayesian linear latent growth models: (a) a Gaussian model, (b) a model based on Student’s *t*-distribution (i.e., a robust model), and (c) an asymmetric Laplace model (i.e., Bayesian quantile regression and an alternative robust model). Based on leave-one-out cross-validation and posterior predictive model checking, we found that both robust models outperformed the Gaussian model, and both robust models performed comparably well. While the Student’s *t* model performed statistically slightly better (yet not substantially so), the asymmetric Laplace model yielded somewhat more realistic posterior predictive samples and a higher degree of measurement precision (i.e., for those estimates that were either associated with the lowest or highest degree of measurement precision). The findings are discussed for the context of learning progress assessment.

## 1. Introduction

The term progress monitoring refers to systematically gathering information on students’ learning progress to guide feedback and instructional decision making. A prominent example of progress monitoring is curriculum-based measurement (CBM; Deno 1985), occurring in the context of special education. In CBM, parallel weekly assessments of core competencies such as reading are used to assess students’ responsiveness to teachers’ instructional decisions. An important feature of CBM is that assessments are indicators of and interpreted in relation to a desired learning goal (Fuchs 2004). Another similar form of progress monitoring is learning progress assessment (LPA), which refers to progress monitoring in everyday classrooms. LPA as implemented by the assessment system quop (Souvignier et al. 2021), for example, has longer time intervals between successive measurement points as compared to CBM. In addition, LPA tries to balance differentiated assessment of relevant skills (i.e., math and reading achievement) to allow differentiated feedback and acceptable psychometric properties. For example, the quop-L2 test series for reading assessment in second grade includes three subscales at all levels of language (i.e., the word, sentence, and text levels; Förster et al. 2021; Förster and Kuhn 2021). If a student performs well at the word level but poorly at the sentence and text levels, the fit of instruction would be high if the teacher supports the student’s sentence reading before supporting more complex higher-order reading comprehension strategies. The success of such progress monitoring implementations (regardless of whether CBM or LPA) can be evaluated via estimates of learning progress (Fuchs 2004).

### 1.1. Estimation of Learning Progress

The idea of estimating learning progress using the slope of a student’s data plotted in a bivariate scatterplot can be traced back to work prior to the emergence of CBM (Deno and Mirkin 1977). Conceptually, the linear slope that occurs when plotting student performance against measurement time points allows one to assess the student’s average learning progress over time (Silberglitt and Hintze 2007). While numerous methods exist for estimating slopes (Ardoin et al. 2013), in the context of progress monitoring, researchers have most often used ordinary least squares estimation. Historically, ordinary least squares can be understood as having replaced other methods such as quarter-intersect or split-middle (both methods require splitting the data into two halves to identify the median of each halve which builds the basis for drawing the slope; split-middle further requires that the same number of points is situated below and above the line) as the default in progress monitoring. Quarter-intersect or split-middle were considered more applicable in early years of CBM practice when computational power was not regularly available in school settings and growth estimates had to be calculated and drawn by hand (Ardoin et al. 2013). In addition, it was demonstrated in simulation studies that ordinary least squares estimates outperform estimates based on the medians of splitted data (Christ et al. 2012). Most recently, researchers have discussed and examined approaches that can be understood as either robust methods (e.g., non-parametric Theil–Sen regression; Bulut and Cormier 2018; Vannest et al. 2012) or Bayesian methods (Christ and Desjardins 2018; Solomon and Forsberg 2017). The ordinary least squares estimator makes assumptions (e.g., homoscedastic normally distributed errors) that can be violated in empirical data, and it is prone to influencing outliers. Indeed, the non-parametric Theil–Sen estimator does not require such strong assumptions and is robust with respect to outliers. Advantages of Bayesian estimation methods have been nicely summarized by Solomon and Forsberg (2017, p. 542): it can be robust, prior information can be utilized, and it has a natural compatibility with data-based decision making (i.e., posterior probabilities inform about intervention success).

Importantly, students may occasionally be tired or unmotivated when taking a test. In addition, researchers have identified that particular factors related to data collection (e.g., the place where the assessment is conducted, the person administering and/or scoring the test) may cause scores to fluctuate (Van Norman and Parker 2018). Hence, in the context of progress monitoring (i.e., repeated assessment of learning progress to inform feedback and instructional decision making), such fluctuations potentially influence performance at single measurement points and might yield single observations that strongly deviate from what might be expected (Bulut and Cormier 2018). Consequently, such outliers can influence estimates of student learning, especially when they occur at the beginning or toward the end of the period of assessment (Bulut and Cormier 2018). However, an accurate evaluation of student learning is critically important in the context of progress monitoring because such a data-based approach to decision making (Espin et al. 2017) relies on dynamic loops of assessment, instructional decisions, and feedback. To avoid this problem, Christ and Desjardins (2018) suggested using Bayesian slope estimation, which was found to be more precise and more realistic compared to ordinary least squares regression (Christ and Desjardins 2018).

### 1.2. Factors That Influence the Quality of Learning Progress Estimates

The quality of slope estimates in the context of progress monitoring does not only depend on the method of slope estimation. Both empirical and simulation studies have identified several other factors affecting the psychometric integrity of slope estimates such as measurement invariance, procedures of data collection, data collection schedules, and the number of measurement points. For a review of these factors from the perspective of CBM, see Ardoin et al. (2013).

Measurement invariance of the tests used is important to allow a straightforward interpretation of learning progress. While Ardoin et al. (2013) concluded that empirical tests of probe equivalence in CBMs are scarce in the literature, the importance of equivalent (i.e., parallel) tests has been emphasized in progress monitoring research. As recommended by Schurig et al. (2021, p. 2): “… a good progress monitoring test should first check the dimensions, then the invariance…”. The available evidence of CBM probes in terms of equivalence suggests that probes may not display form equivalence (Cummings et al. 2013) and findings indicated that psychometric quality of slope estimates depends on the chosen probe sets (Christ and Ardoin 2009). However, the quop-L2 test that was used in this work has demonstrated its factorial validity (Förster et al. 2021) and strong evidence in terms of practical equivalence based on a thorough item-response theory investigation focusing on accuracy and speed (Förster and Kuhn 2021), as well as strict measurement invariance when items are scored for efficiency of reading (Förster et al. 2021). The relevance of procedures of data collection has been already discussed in the introduction above. Clearly, variations in administration procedures can cause fluctuations in test performance, starting with varying times on the testing day at which tests are administered to a simple change of the testing room, for example. Beyond such potential influences on test performance, Bulut and Cormier (2018) thoroughly discussed progress monitoring schedules and the overall number of assessment points. They highlight that optimal schedules depend on the expected rate of improvement, which in turn can depends on various student characteristics. For example, from the perspective of CBM, a comprehensive simulation study revealed that validity and reliability of slope estimates depend on the overall duration (i.e., in weeks) of progress monitoring as well as the number of assessments within each week (Christ et al. 2013). Christ et al. (2013) found that valid and reliable slope estimation required at least four weeks of progress monitoring. While the overall duration of progress monitoring in LPA tends to be longer (e.g., 31 weeks in this study), the overall schedule must be considered to be clearly less dense with successive measurement timepoints being separated by approximately three-week intervals (Souvignier et al. 2021), for example. Beyond these aspects of the progress monitoring schedule, increasing the number of measurement points will increase the measurement precision of slope estimates. However, adding measurement timepoints close in time will, for most core skills, not result in huge information gains when it comes to slope assessment.

### 1.3. Aim of the Current Study

In this work, we aimed at employing robust Bayesian regression based on Student’s *t*-distribution (Kruschke 2015) and Bayesian quantile regression (Yu and Moyeed 2001) to model the conditional median, as these approaches represent promising alternatives following both Christ and Desjardins’s recommendation to use Bayesian estimation and Bulut and Cormier’s call for robust slope estimation when outliers are present (Bulut and Cormier 2018). Importantly, estimating learning progress has relied on approaches that estimate progress for each student separately, but researchers have also applied overarching model frameworks, such as latent growth modeling, to progress monitoring data (Schatschneider et al. 2008; Yeo et al. 2012). Thus, the aim of this work was to combine robust and Bayesian learning progress estimation (i.e., robust Bayesian learning progress estimation) and extend these two recent lines of research toward the framework of linear latent growth models.

In this work, we explored the following research questions in the context of learning progress assessment of reading achievement in the second grade:

**Research Question 1:** Do robust Bayesian latent growth models outperform a simple Bayesian latent growth model based on the Gaussian distribution in terms of learning progress estimation?
**Research Question 2:** Which robust Bayesian latent growth model performs best in terms of learning progress estimation?

To answer these questions, we fitted Bayesian linear latent growth models based either on a Gaussian or a Student *t*-distribution to model reading comprehension efficiency. In addition, we added a Bayesian latent growth model based on an asymmetric Laplace model (i.e., Bayesian quantile regression) with the median as a conditional quantile to the set of candidate models.

## 2. Materials and Methods

### 2.1. Dataset

The dataset we used for this study was also used in a recent study on the reliability of learning progress estimates (Forthmann et al. 2021). In this dataset, *N* = 4970 second-grade students (age in years: *M* = 7.95, *SD* = 0.48; 53% boys and 47% girls) were assessed with the quop-L2 test series for reading achievement (Förster et al. 2021), quop-L2 comprising of equivalently constructed reading tests at all levels of language (i.e., the word, sentence, and text levels). Tests were administered at eight time points throughout the school year 2018/2019 via the assessment system quop (Souvignier et al. 2021). All tests were administered with about three-week intervals between successive tests starting in fall 2018, with two tests prior to the Christmas break. After Christmas break, four tests were administered prior to the Easter break in 2019 and the last two tests were administered between Easter break and Summer break. Thus, in total, the tests were completed over a period of 31 weeks. Initially, the cohort comprised of 6000 students. A total of 1030 students were excluded for the following reasons: (a) 140 students from international schools, (b) 227 students who were not in second grade but assigned to quop-L2, (c) three students who were younger than six years, (d) 94 students who were older than twelve years, (e) 333 students who had missing values on all measurement points, and (f) 233 identified duplicate cases.

For each item, subscale-specific quantiles were used as cut-offs for valid response behavior to correct for fast guessing (Wise 2017; Wise and DeMars 2010) and inacceptable slow responding. For fast guessing we used the 5%-quantile, whereas for slow responding we used the 99.5%-quantile. These quantiles were calculated for the complete sample comprising of all cohorts from 2015 to 2019 (*N* = 15,700) and for each subscale across all items (word level: lower bound = 1362.98 ms, upper bound = 41,032.86 ms; sentence level: lower bound = 1427.02 ms, upper bound = 53,742.18 ms; text level: lower bound = 877.36 ms, upper bound = 85,836.71 ms). These cut-offs were used prior to accuracy scoring of the items. In addition, the correct item summed residual time (CISRT) scoring was used as a measure of efficiency (Maris and van der Maas 2012). The time cut-offs were also useful for CISRT scoring which required item timing and quop-L2 tests are administered without any acute time limits. Item CISRT scores were averaged for each subscale (i.e., word, sentence, and text level) and scaled to be in the range from 0 to 10. Notably, a scoring of efficiency was used for two reasons: (a) it is in accordance with developmental models and empirical findings on reading skills, and (b) it allows assessing individual differences even among highly-proficient students. Developmental models of reading suggest that reading accuracy develops earlier, prior to automatizing the process into fluent reading (i.e., accurate and quick reading; Juul et al. 2014). The CISRT scoring mimics this by awarding fast reading only when the process was accurate. In addition, ceiling effects are likely for highly-proficient students in regular classes, when only accuracy is scored. Hence, introducing an additional speeded component or a scoring for efficiency for reading assessment is vital to measure individual differences in regular classrooms (Forthmann et al. 2020).

In this work, the more general construct of reading achievement was the focus (i.e., the higher order construct of reading at the word, sentence, and text level). Hence, scores at the word, sentence, and text level were used as observed indicators in a latent variable model to establish strong measurement invariance (Vandenberg and Lance 2000) prior to growth modeling. First, we established a configural model by comparing a simple longitudinal confirmatory factor analysis (CFA) model with one latent reading achievement variable based on the three observed scores at word, sentence, and text level at each of the eight timepoints. All latent covariances of the model were freely estimated, but residual covariances of the observed scores were fixed to a value of zero (Model 1). The loading of the word level indicator was fixed to a value of one at each timepoint to identify the model. We compared this configural model with another model that allowed residual covariances between scores at the same level. For example, all residual covariances between word-level scores were freely estimated, but cross-level covariances (e.g., between word-level and sentence-level scores) were fixed to zero (Model 2). As it turned out that residual covariances for sentence-level scores were mostly non-significant and rather small in size, we decided to add a more data driven and more parsimonious model that incorporated only residual covariances for word-level and text-level scores (Model 3). All models were estimated by the lavaan package (Rosseel 2012, version 0.6-9) for the statistical software R (R Core Team 2021, version 4.1.2 used on a local computer). We used full information maximum likelihood for model estimation to take missing values into account. This is justified by the fact that for this kind of longitudinal data, missing at random is the most likely underlying missing data mechanism (Asendorpf et al. 2014) and previous analyses of the missing data in quop-L2 also revealed patterns in accordance with missing at random (Förster et al. 2021). Robust maximum likelihood estimation was used to account for non-normality of the data. For general recommendations, with respect to model fit indices, we refer to relevant textbook chapters (West et al. 2012). For measurement invariance models we used the established criterion that the CFI should not decrease by more than .010 and complement change in CFI by change in RMSEA and SRMR (Chen 2007). Model 3 was chosen as the configural model based on highly comparable findings (Model 1: CFI = .920, RMSEA = .065, SRMR = .041; Model 2: CFI = .996, RMSEA = .019, SRMR = .010; Model 3: CFI = .994, RMSEA = .020, SRMR = .013). The decrease in CFI from this configural model to a strong invariance model (i.e., loadings and intercepts of the scores are constraint to be equal across time) was −.006, which was clearly smaller than the .010 criterion. In addition, this was accompanied by an increase of .007 for the RMSEA and .022 for the SRMR. We concluded that strong measurement invariance across time was quite reasonable for reading achievement measured by the subscales of quop-L2.

Next, we extracted factor scores by means of the Bartlett method (DiStefano et al. 2009) from the strong invariance model. To empirically justify the use of factor scores, we examined factor determinacy indices (FDI; Ferrando and Lorenzo-Seva 2018). FDIs were all excellent (all FDIs > .90) and allowed usage of factor scores even for individual assessments (Ferrando and Lorenzo-Seva 2018). These factor scores were the dependent variable (i.e., *y_pt_*) in the latent growth models, as defined in Table 1. Hence, we have used a scoring based on standard maximum likelihood CFA in a first step and used Bayesian latent growth models in a second step. It should be mentioned that factor scores could be calculated for all students at all measurement timepoints. Hence, no missing values were present in the data when the latent growth models were estimated (see Section 2.2 below). We further estimated reliability by means of Cronbach’s α (Cronbach 1951) and Bollen’s ω_1_ (Bollen 1980; Raykov 2001), as implemented in the semTools package (Jorgensen et al. 2021, version 0.5-5). Reliability estimates across measurement points were rather homogeneous (Cronbach’s α: range from .74 to .78; Bollen’s ω_1_: range from .74 to .78). Hence, reliability of efficiency scores were clearly above the commonly cited .70 which is required for low-stakes decisions (Christ et al. 2005).

Finally, to make the comparisons presented in this work worthwhile, we examined if outliers were actually present in the data. Based on Mahalanobis distance (Tabachnick and Fidell 2005), we identified *n* = 229 students as multivariate outliers (i.e., approximately 5% of the total sample).

### 2.2. Analytical Approach

All models were fitted with the brms package (Bürkner 2017, 2018) for the statistical software R (R Core Team 2021). All models were estimated on the computer cluster of of the University of Münster (https://www.uni-muenster.de/IT/services/unterstuetzungsleistung/hpc/; accessed on 20 February 2022), and all distributions needed for the current research (Gaussian, Student’s *t*, and asymmetric Laplace distributions) were implemented in brms. For each of the distributions, a linear growth model was specified in the brms model formula syntax. Specifically, the eight measurement points were coded using the numbers 0 to 7, which allowed for interpreting the intercept in the model as the initial value of reading efficiency. Hence, slope estimates represent the average progress after a three-week interval. Average reading progress between successive measurement points was represented in the model by the parameter $\mu_{\beta_{1}}$. Intercept and slope were further modeled as latent variables to allow variation of initial level and learning progress across students. The correlation between intercepts and slopes was also estimated. The exact definitions and used priors for all three models are provided in Table 1.

After a first round of estimating the models, we found that the scale of the factor scores with an average close to zero was problematic for the estimation process. Specifically, we observed non-converging chains as flagged by $\hat{R}$ values around 1.50 and very, very low Bulk-ESS and Tail-ESS measures (all values were far below the recommended cut-offs; Vehtari et al. 2021). This was always observed for the asymmetric Laplace model and occasionally for the Student’s *t* model. First, we experimented with increasing the number of iterations, but convergence issues as well as divergent transitions were still observed. Divergent transitions indicate that the MCMC algorithm cannot be trusted, and that the posterior distribution has not been well sampled (see here https://mc-stan.org/docs/2_19/reference-manual/divergent-transitions; accessed on 18 February 2022). Hence, we decided to simply multiply all factor scores by 30 (i.e., all factor scores at all timepoints) to update the fitted models (this value was arbitrarily increased after an initial attempt to multiply by ten, which indicated that scaling the values this way facilitated model estimation). In addition, we used 4000 iterations for the Gaussian growth model, whereas for the Student’s *t* and asymmetric Laplace models, we needed 6000 iterations. We ran four chains for each model. In addition, for the Student’s *t* and asymmetric Laplace models, we had to set the control parameters max_treedepth and adapt_delta to values of 15 each and 0.95 or 0.90, respectively, to prevent divergent transitions.

Researchers have argued to pay close attention to various convergence diagnostics such as measures of potential scale reduction (PSR) and effective sample size to insure accurate Bayesian inference (e.g., Vehtari et al. 2021; Zitzmann and Hecht 2019). In our work, we used the improved $\hat{R}$ (i.e., PSR), Bulk-ESS, and Tail-ESS convergence statistics proposed and studied by Vehtari et al. (2021). These measures are implemented in Stan (Carpenter et al. 2017), on which brms (Bürkner 2017) is based and is immediately available in the model outputs. $\hat{R}$ should be <1.01 and all of our obtained $\hat{R}$ values were 1.00 (i.e., for all parameters in all models). In addition, Vehtari et al. (2021) recommend the ESS measures to be >400 when four chains are used (which we did for all models). The recommended value of 400 was surpassed for the Gaussian model (range of Bulk-ESS: 1296 to 4141; range of Tail-ESS: 1971 to 3470), the Student’s *t* model (range of Bulk-ESS: 1497 to 9841; range of Tail-ESS: 2811 to 10,181), and the asymmetric Laplace model (range of Bulk-ESS: 1065 to 7590; range of Tail-ESS: 2264 to 8590).

Estimated model parameters were reported, along with 95% credible intervals. In brms, these intervals are based on the respective quantiles of the posterior samples. Cross-validation can be used for Bayesian multi-model inference (Sivula et al. 2020). Hence, models were compared based on approximate leave-one-out cross-validation (LOO; Vehtari et al. 2017). Approximate LOO was performed by Pareto smoothed importance sampling. We used the expected log-pointwise predictive density (ELPD; Vehtari et al. 2017) to evaluate the models’ predictive accuracy. The ELPD difference and its standard error allow a profound evaluation of differences in terms of model fit (i.e., the difference can be interpreted in relation to the standard error).

Finally, to better understand differences between the models, graphical checks and correlational analyses were conducted. For all three models, we examined posterior predictive checking (Gelman et al. 2013). In addition, we looked at the densities of the Gaussian, Student’s *t*, and asymmetric Laplace distributions based on the estimates obtained for the first measurement point of an average student; then, we checked the correlations between the estimates for the initial level and the learning progress based on the different models, and we compared estimates of measurement precision for the initial level and the learning progress between both robust approaches.

## 3. Results

### 3.1. Model Comparison and Model Parameter Findings

We found that the Student’s *t* model performed best, as indicated by the LOO comparison results (i.e., as indicated by the value of zero for the ELPD difference; see Table 2). Notably, the ELPD difference between the Student’s *t* and the asymmetric Laplace models was not larger in absolute size than twice its standard error (see Table 2), showing that both robust approaches performed equally well. The ELPD difference between the model based on Student’s *t*-distribution and the simple Gaussian model was larger in absolute size than sixteen times its standard error, which represents very strong evidence in favor of the robust linear model. Given that the ELPD difference was immense when comparing the Student’s *t* and the Gaussian models and negligible when comparing the Student’s *t* and the asymmetric Laplace models, we concluded that both robust methods clearly outperform the Gaussian model.

In addition, the latent variable results were also more comparable when comparing both robust methods, and less similar for when each robust method was compared to the Gaussian model. At the same time, the overall pattern of findings was quite comparable. In terms of mean vectors, we found negative intercepts in all models (see Table 2), and the slope estimates were highly similar across all models. For the latent variable results (i.e., between person variation results), we found in all models that there were larger standard deviations for the intercepts than for the slopes. This hints at stronger interindividual differences in the initial level of reading efficiency as compared to interindividual differences in average learning progress between successive measurement points. The correlation between random intercepts and slopes was negative and large in size (see Table 2). Hence, children with higher initial levels tended to make less learning progress over the school year in terms of reading efficiency. Finally, the models imply different levels of within-person variation as indicated by the residual variance estimates. Residual variance was highest for the Gaussian model and smallest for the Asymmetric Laplace model (see Table 2).

### 3.2. Exploring Differences between the Models

We found that both robust modeling approaches performed similarly well in terms of the LOO comparison results (see Table 2). However, the Student’s *t* and asymmetric Laplace distributions have different properties, implying differences between both models that deserve further exploration. For illustration and to facilitate a deeper understanding of the reported findings, in this section we also consider results for the Gaussian distribution.

**Figure 1:**
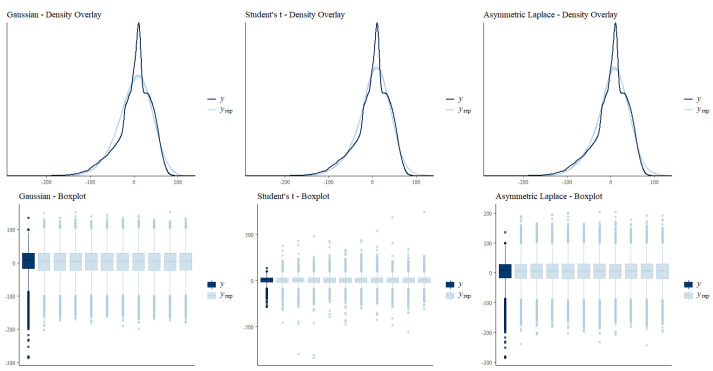
Graphical posterior predictive checking results. Top: Density overlay (based on ten posterior draws for each of the models) restricted to the range of −290 to 140 on the *x* axis to facilitate a comparison of model fit based on the main part of the empirical distribution (i.e., observed values of reading efficiency *y* ranged from −286.11 to 135.88). Bottom: Boxplots of the original data (dark blue) and ten draws of the posterior predictive distribution (boxes in light blue) to facilitate comparison of the sampled values between the three models.

Looking at graphical posterior predictive checking results (see Figure 1), it became apparent that both robust approaches were better able to model the peak around zero of the distribution of reading efficiency compared to the Gaussian model (see top row of plots in Figure 1). Upon visually inspecting the densities of observed (dark blue line) and sampled (light blue lines) reading efficiency within the range of observed values (the reading efficiency factor scores multiplied by 30 ranged from −286.11 to 135.88), we found no differences between both robust approaches in terms of behavior of predictive posterior samples (see top-middle and top-right plots in Figure 1).

Differences between both robust approaches only became visible when looking at the full ranges of predictive posterior samples (light blue boxes and dots), as depicted in the plots in the bottom row of Figure 1. Posterior predictive samples based on Student’s *t*-distribution had ranges that well covered all outliers at the lower tail of the distribution of reading efficiency (for some draws, the lower-tail outliers were nicely replicated). However, the range of sampled values was clearly wider than the observed range of values; this was particularly the case for the upper tail of the distribution (see bottom-middle plot in Figure 1). Hence, the model based on Student’s *t*-distribution produced outliers that strongly exceeded the most extreme cases of the observed data. Regarding the asymmetric Laplace model, this model was able to cover a portion of the extreme values at the lower tail of the distribution of reading efficiency factor scores (see bottom-right plot in Figure 1), but the most extreme values were not fully covered. At the same time, posterior predictive samples based on the asymmetric Laplace distribution did not exceed the observed values as much as they did for Student’s *t* at the upper tail. Hence, one might conclude that the asymmetric Laplace model yielded somewhat more realistic posterior predictive samples than the Student’s *t* model did.

The Gaussian model (bottom-left plot in Figure 1) did not cover any of the most extreme values at the lower tail of the distribution of reading efficiency and—compared to both robust approaches—the box width of the original data was less well reproduced (this fact was harder to inspect by eye when looking at the density overlay plots in the top row of Figure 1). Consequently, posterior predictive checking provided further insights into the question of why both robust models performed better than the Gaussian model (i.e., they better reproduced the box width of the original data and better reproduced extreme values at the lower tail of the distribution) and how both robust approaches actually differed (i.e., the Student’s *t* model better covered extreme values at the lower tail of the distribution, whereas the asymmetric Laplace model seemed to produce posterior predictive samples within the range of the observed values of reading efficiency).

**Figure 2:**
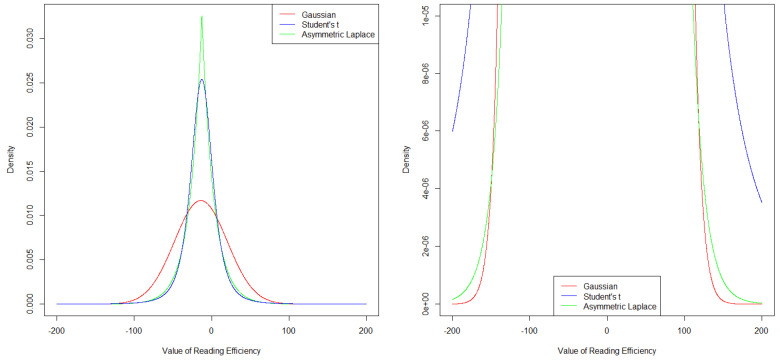
Distribution density plots for the following distributions (cf. Table 2): *N*(−13.98, 34.10), *t* (−12.79, 14.55, 3.22), and *ALD* (−12.70, 7.67, 0.50). These are the distributions based on the estimated models reported in Table 2 for reading efficiency at the first measurement point for the average student. Left: *y*-axis range from 0 to 0.035 and *x*-axis range from −200 to 200. Right: “zoom-in” depiction of the densities to better visualize differences at the tails, i.e., *y*-axis range from 0 to 0.00001 and *x*-axis range from −200 to 200.

We further examined the densities of the distributions, as based on the models reported in Table 2 for the first measurement point of an average student, to further understand how both robust models were able to better model the peak (and box width) of the original data distribution. The densities are depicted in Figure 2, where the left side of Figure 2 displays the full densities. Both robust models had more strongly peaked densities at the center of the distributions. In addition, the densities at the tails of the robust distributions had more probability mass than did those of the Gaussian distribution (see the right side of Figure 2). This explains exactly why these distributions work better when extreme values are present in the data: Such extreme values have a higher likelihood under these models and, hence, will have less influence on the model results as compared to the Gaussian model. This difference at the tails was clearly more pronounced for the Student’s *t* model than the asymmetric Laplace model, which again suggests that the Student’s *t* model was better able to handle the observed extreme values at the lower tail of the reading efficiency distribution (cf. Figure 1).

**Figure 3:**
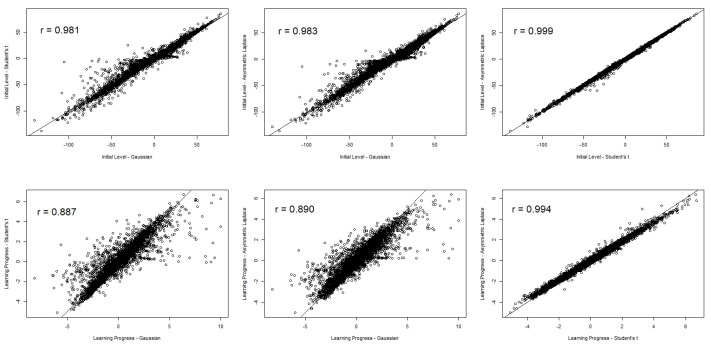
Bivariate scatter plots between initial level and learning progress estimates.

Next, we examined the correlations between the initial level (i.e., the random intercept estimates) and the learning progress estimates (i.e., the random slope estimates) between the three models (see Figure 3). These correlations allowed us to check whether the relative positioning of students with respect to important progress monitoring information differed between the models. The correlations between the initial level estimates are depicted in the top row of Figure 3; the initial level estimates based on both robust models correlated almost perfectly with the estimates obtained from the Gaussian model. In addition, initial level estimated based on both robust models revealed a perfect correlation (see top-right plot in Figure 3). Thus, estimating the initial level was quite robust across the three studied models. However, when looking at learning progress estimates, model choice clearly mattered for the relative positioning of students. The correlation between estimates based on the Gaussian model and both robust models was still large, but it was substantially lower (*r* ≈ .89) than the correlation between learning progress estimates obtained from both robust models (the correlation was nearly perfect; see bottom-right plot in Figure 3). Thus, the differences between the Gaussian model and both robust models in terms of relative positioning were more strongly pronounced for learning progress than for initial level estimates, whereas both robust models yielded perfectly correlated estimates in this regard.

**Figure 4:**
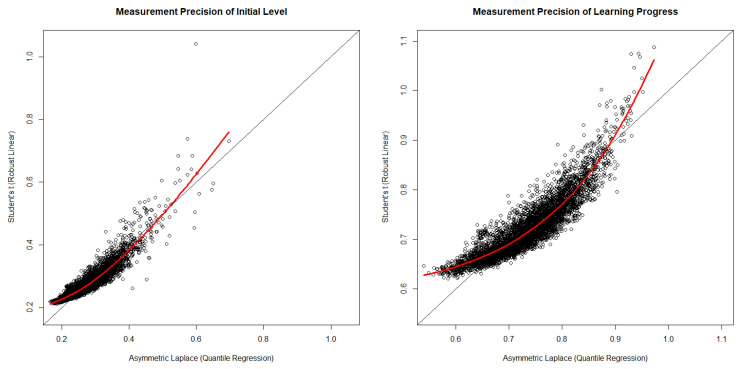
Bivariate scatter plots of measurement precision estimates. Red line = LOESS curve.

Finally, we investigated the measurement precision (i.e., the standard deviations of the posterior samples) of the initial level and the learning progress estimates based on both robust models. As both robust models make different distributional assumptions, the estimates of measurement precision for the initial level and the learning progress depended on the characteristics of these distributions (e.g., the asymmetric Laplace distribution was more peaked, whereas Student’s *t*-distribution had heavier tails; see Figure 2 above). To compare measurement precision between both robust models, we divided the standard deviations of the posterior samples of initial level and learning progress estimates by their respectively estimated standard deviations of the respective latent variable distributions reported in Table 2. As depicted in Figure 4, we found that the measurement precision of the initial level estimates was clearly higher than the measurement precision of the learning progress estimates (compare the left and right plots in Figure 4). In addition, estimates of the measurement precision for both models had a positive yet non-linear relationship (see the LOESS curves depicted in red in both plots in Figure 4). At the lower and upper tails of measurement precision estimates, we observed that the measurement precision based on the asymmetric Laplace model was higher. Hence, in situations that allow for choosing an asymmetric Laplace latent growth model, learning progress parameters for individual students (i.e., those estimates that tend to be associated with the lowest or highest measurement precision) would be expected to have greater measurement precision than when applying Student’s *t*-distribution.

## 4. Discussion

In this work, we examined three Bayesian models to estimate student learning progress to extend recent calls for using Bayesian and robust approaches (Bulut and Cormier 2018; Christ and Desjardins 2018; Solomon and Forsberg 2017). We found that both robust models had better model-data fit than a Gaussian linear growth model did. Christ and Desjardins (2018) found that Bayesian Gaussian linear regression worked better than standard ordinary least squares regression for estimating the learning progress of individual students. Here, however, latent growth models were estimated and compared with each other. We found that linear growth models based on the Student’s *t*-distribution and the asymmetric Laplace distribution performed very similarly in terms of LOO comparison and also in terms of relative positioning of students with respect to the initial level and the learning progress estimates, as revealed by nearly perfect correlations between the estimates of these two models. In addition, we showed that both robust models were better than the Gaussian model at modeling the peak and lower tail of the distribution of reading efficiency. Specifically, the likelihood of extreme values at the lower end of the tail of the distribution was higher for both robust models, meaning that these values are not as influential on model estimation as they are for the Gaussian model (Kruschke 2015). Indeed, this behavior was more pronounced for the Student’s *t* model. However, differences between these two models were also revealed. While the Student’s *t* model performed statistically slightly better (in terms of LOO comparison and posterior predictive samples that well covered extreme values at the lower tail of the reading efficiency distribution), the asymmetric Laplace model yielded somewhat more realistic posterior predictive samples and gave a higher degree of measurement precision for those estimates that were associated with either the lowest or highest degrees of measurement precision.

These findings may generalize to other core competencies (e.g., math skills; Salaschek and Souvignier 2014; Boorse and Van Norman 2021), grade levels (e.g., reading comprehension in fourth grade; Förster and Souvignier 2014), and contexts (e.g., special needs educational setting; Jenkins et al. 2009) as long as the score distribution shows signs of being more peaked or having more extreme values at the tails of the distribution of progress monitoring data. However, we recommend that all three models be carefully examined in terms of model fit because the models have different distributional characteristics that imply, for example, differences in the measurement precision of learning progress monitoring estimates. Hence, the choice of model should be guided empirically, not based on favorable model properties per se (i.e., without any evaluation of model fit). Another option with this particular set of candidate models would be to test whether the conclusions drawn from analyses are robust across the models.

Despite differences between CBM and LPA (see Section 1), there are common aspects also. The estimation of learning progress (i.e., growth) by means of slopes, for example, is a technical feature that is shared by CBM and LPA. We are well aware that the difference in schedules has implications for the quality of slope estimates. However, we are confident that our study has implications even for CBM growth modeling, despite these differences. First, while CBM research on slope estimates often focuses on single-case data (e.g., Christ and Desjardins 2018; Solomon and Forsberg 2017), there are also latent growth model applications in the CBM literature (Keller-Margulis and Mercer 2014; Yeo et al. 2012). Importantly, for these applications, even larger intervals between successive measurements were allowed (e.g., testing in fall, winter, and spring) as compared to the ones used in our sample (approximately three-week intervals). Hence, given that CBM displays quite a range in terms of intervals between successive measurement points, our work has implications for such latent growth model applications within the CBM framework (i.e., at least when we consider potential differences in terms of inter-test intervals).

Beyond these potential differences (and similarities) between CBM and LPA applications of progress monitoring, however, we would like to highlight that the proof-of-concept provided by the empirical findings in our work are most likely to generalize when outliers are present in progress monitoring data. In our dataset we found that approximately 5% of the students qualified as multivariate outliers (this was further evident in the visual inspections of the data; see Figure 1). It is hard to say if this percentage of extreme cases is representative for progress monitoring data in general. However, for datasets with a larger number of extreme cases, it is clear that the current findings matter (perhaps even more). In addition, even for less influential cases, our work has clear implications. That can be easily seen when looking at slope estimation of single-case data that can be heavily influenced by only one influencing outlier at one of the measurement points (Bulut and Cormier 2018). Using a model based on Student’s *t* distribution instead of a Gaussian model will be a much better choice in that situation as compared to a Gaussian model. In relation to this, Christ and Desjardins (2018) have examined and discussed the role of prior choice and concluded that choosing reasonable priors is crucial for Bayesian slope estimation to have an advantage in terms of measurement precision and realistic estimates over ordinary leas squares regression. Choosing reasonable priors might only result when knowledge about the distributions of both intercept and slope is available to inform Bayesian slope estimation of single-case data. Specifically, knowledge that is obtained from latent growth models applied to a large progress monitoring dataset, such as the one studied in this work, seems to be particularly useful to construct reasonable priors for single-case estimation. Otherwise, it has been shown that such type of knowledge is not always needed to construct advantageous priors (Finch and Miller 2019; Zitzmann et al. 2021). For instance, Zitzmann et al. (2021) showed analytically that priors for variability parameters can perform well even when they are incorrect and do not represent the “true” parameter. Future research is needed to investigate such phenomena for robust Bayesian growth models in the context of progress monitoring.

This research was limited to reading comprehension in second-grade regular classrooms. In addition, we operationalized reading comprehension as a higher-order construct, but the quop-L2 test series also allows for a more detailed look at reading comprehension at the word, sentence, and text levels. Given that these subdimensions of quop-L2 might have different empirical distributions, it is not clear whether the findings generalize to each of the test’s subdimensions. It should further be mentioned that we considered only linear growth models, as they fit nicely with the traditional conception of learning progress in the CBM literature (Silberglitt and Hintze 2007). However, researchers have also examined more complex growth models such as quadratic growth (Schatschneider et al. 2008). Hence, beyond the distribution of the used growth model, we consider it a promising path for future research to further compare different functional forms of the growth trajectory.

Notably, we used a scoring based on standard maximum likelihood CFA in a first step and used Bayesian latent growth models in a second step. While this might appear overly pragmatic in the sense that we did not hesitate to cross two different statistical philosophies within one and the same analysis, we are convinced that there are good reasons to believe that choosing this two-step approach has not undermined our aims. First, this initial step was undertaken to control for potential differences with respect to psychometric properties across timepoints (e.g., differences in intercepts of word, sentence, and text level scores could have influenced learning progress estimates within the latent growth curve model). The alternative would have been to use sum scores of observed scores, for example, which cannot be assumed to be of the same psychometric quality. Second, approximately 5% of the participants were identified as multivariate outliers which emphasizes the general need for robust modeling. Third, in the tradition of progress monitoring research, outliers are considered at the level of scores at each of the measurement timepoints (e.g., Bulut and Cormier 2018). The outliers in our work were also considered and taken into account by robust Bayesian latent growth models at the level of scores at different timepoints. One-step approaches in which latent variables are modeled at each measurement point and growth is modeled by means of higher-order latent variables would have shifted the question of outliers to the level of indicators of reading efficiency at each timepoint (one could even think of models in which the focus is shifted to item-level outliers). We argue that such a shift of the focus of outlier treatment would be worth future investigations, but it was beyond the aims of our current investigation.

Moreover, it should be discussed that we needed to fit all models on a high-performance computer cluster. A simple desktop or laptop would not have succeeded in this task within the same amount of time. Hence, while Bayesian model syntax building and model specification is clearly facilitated by the user-friendly implementation of brms (Bürkner 2017), special knowledge to run these models on a computer cluster is needed to make this workable for a large progress monitoring dataset, as studied in this work.

To conclude, the current work adds to the literature on learning progress estimation by combining the ideas of robust and Bayesian estimation into an overarching latent growth modeling framework. Here we showed that robust latent growth models outperform standard Gaussian models, and we found that these models were better capable of modeling a stronger peaked distribution and more extreme values at the lower tail of the distribution. We further found that the asymmetric Laplace model had, for some estimates, a higher degree of measurement precision, and yielded more realistic posterior predictive samples. These findings look promising for future applications, and we hope that the outlined methods in this work will be tested and extended in the field of progress monitoring research.

## Figures and Tables

**Table 1:** Linear Latent Growth Model Definitions and Used Prior Distributions.

Model	Gaussian	Student’s *t*	Asymmetric Laplace
Response Distribution	$y_{pt}\;\sim \;N\left(\eta_{pt},\;\sigma^{2}\right)$	$y_{pt}\;\sim \;t\left(\eta_{pt},\;\sigma^{2},\;\nu \right)$	$y_{pt}\;\sim \;ALD\left(\eta_{pt},\;\sigma^{2},\;0.50\right)$
Linear Predictor	$\eta_{pt}=\beta_{0,p}+\beta_{1,p}X_{t}$	$\eta_{pt}=\beta_{0,p}+\beta_{1,p}X_{t}$	$\eta_{pt}=\beta_{0,p}+\beta_{1,p}X_{t}$
Latent Variable Distribution	$\;\beta_{p}\;\sim \;MVN\left(\mu_{\beta },\Sigma_{\beta }\right)$	$\beta_{p}\;\sim \;MVN\left(\mu_{\beta },\Sigma_{\beta }\right)$	$\beta_{p}\;\sim \;MVN\left(\mu_{\beta },\Sigma_{\beta }\right)$
$\mathrm{Prior}\;\mathrm{for}\;\mu_{\beta_{0}}$	t(0.3,2.5,3)	t(0.3,2.5,3)	t(0.3,2.5,3)
$\mathrm{Prior}\;\mathrm{for}\;\mu_{\beta_{1}}$	Improper flat prior	Improper flat prior	Improper flat prior
$\mathrm{Prior}\;\mathrm{for}\;\sigma_{\beta_{0}}$	ht(0,2.5,3)	ht(0,2.5,3)	ht(0,2.5,3)
$\mathrm{Prior}\;\mathrm{for}\;\sigma_{\beta_{1}}$	ht(0,2.5,3)	ht(0,2.5,3)	ht(0,2.5,3)
Prior for correlation matrices	lkj(1)	lkj(1)	lkj(1)
Prior for *σ*	ht(0,2.5,3)	ht(0,2.5,3)	ht(0,2.5,3)
Prior for *ν*	-	Γ(2,0.10)	-

$y_{pt}$ = reading efficiency factor score for person *p* at timepoint *t*. $\eta_{pt}$ = linear predictor for person *p* at timepoint t. $\sigma^{2}$ = Residual variance. ν = degrees of freedom of Student’s *t*-distribution. $\beta_{0,p}$ = Intercept of person *p* (i.e., initial level of reading efficiency). $\beta_{1,p}$ = Slope of person *p* (i.e., learning progress in reading efficiency). $X_{t}$ = Coding variable of measurement timepoint *t* ($X_{1}=0,X_{2}=1,\ldots ,X_{8}=7)$. $\beta_{p}$ = Matrix of latent variables $\beta_{0,p}$ and $\beta_{1,p}$. $\mu_{\beta }$ = Vector of latent variable means $\mu_{\beta_{0}}$ (i.e., the average intercept across all persons) and $\mu_{\beta_{1}}$ (i.e., the average slope across all persons). $\Sigma_{\beta }$ = Covariance matrix of latent variables $\beta_{0,p}$ and $\beta_{1,p}$. *N*() = Normal distribution. *t*() = Student’s *t* distribution. *ALD*() = Asymmetric Laplace distribution. *MVN*() = Multivariate normal distribution. *ht*() = Half-*t* distribution. *lkj*() = Lewandowski-Kurowicka-Joe distribution. Γ() = Gamma distribution.

**Table 2:** Model Estimates and Comparisons for the Latent Growth Curve Models.

Model	Gaussian		Student’s *t*		Asymmetric Laplace	
	Estimate	95% CI	Estimate	95% CI	Estimate	95% CI
Person Level (Latent Variables)						
${\hat{\sigma }}_{\beta_{0}}$	34.10	[33.32, 34.90]	34.28	[33.48, 35.06]	34.17	[33.38, 34.98]
${\hat{\sigma }}_{\beta_{1}}$	2.79	[2.65, 2.94]	2.41	[2.28, 2.54]	2.29	[2.15, 2.42]
$\mathrm{Cor}(\beta_{0,p},\beta_{1,p}$)	−0.55	[−0.58, −0.52]	−0.63	[−0.67, −0.60]	−0.63	[−0.66, −0.59]
Population Level						
$\mu_{\beta_{0}}$	−13.98	[−15.00, −12.96]	−12.79	[−13.79, −11.75]	−12.70	[−13.74, −11.68]
$\mu_{\beta_{1}}$	4.52	[4.40, 4.64]	4.59	[4.49, 4.70]	4.57	[4.46, 4.67]
*σ*	21.90	[21.73, 22.08]	14.55	[14.31, 14.80]	7.67	[7.59, 7.76]
*ν*	-	-	3.22	[3.08, 3.36]	-	-
Quantile	-	-	-	-	0.50	-
LOO Comparison						
ELPD Difference	−2600.60		0.00		−32.50	
ELPD Difference *SE*	154.00		0.00		30.40	

CI = credible interval. LOO = leave-one-out cross-validation. ELPD = expected log-pointwise predictive density. SE = standard error. Please see Table 1 for model definitions and equations.

## Data Availability

The analyses scripts and data used for this study are openly available in the Open Science Framework: https://osf.io/hjx43/ (accessed on 18 February 2022).
